# Supplementary material for: Improving pathway prediction accuracy of constraints-based metabolic network models by treating enzymes as microcompartments
Source: Synth Syst Biotechnol. 2023 Sep 12;8(4):597–605. doi: 10.1016/j.synbio.2023.09.002 (PMC10514394; doi:10.1016/j.synbio.2023.09.002)
Supplement: Multimedia component 1 [file mmc1.docx]

**Supplementary material**

**Table S1** Thermodynamic bottleneck reaction(s) in the l-serine synthesis pathways

| **Turning** | **Max flux** | **MDF** | **Bottleneck** | **Reaction equation** |
| --- | --- | --- | --- | --- |
| **point** | **(mmol/gDW/h)** | **(kJ/mol)** | **reaction** |  |
| 1 | 5.681 | 9.864 | ACONTa | cit_c --> acon_C_c + h2o_c |
|  |  |  | ACONTb | acon_C_c + h2o_c --> icit_c |
| 2 | 5.916 | 9.358 | PGI_r | f6p_c --> g6p_c |
|  |  |  | RPE | ru5p__D_c --> xu5p__D_c |
|  |  |  | G6PDH2r | g6p_c + nadp_c --> 6pgl_c + h_c + nadph_c |
|  |  |  | TALA | g3p_c + s7p_c --> e4p_c + f6p_c |
|  |  |  | TKT2 | e4p_c + xu5p__D_c --> f6p_c + g3p_c |
|  |  |  | TKT1 | r5p_c + xu5p__D_c --> g3p_c + s7p_c |
|  |  |  | TPI_r | g3p_c --> dhap_c |
|  |  |  | FBA_r | dhap_c + g3p_c --> fdp_c |
| 3 | 5.923 | 7.638 | ADK1 | amp_c + atp_c --> 2.0 adp_c |
| 4 | 8.306 | 7.422 | PGK_r | 13dpg_c + adp_c --> 3pg_c + atp_c |
|  |  |  | PGM_r | 3pg_c --> 2pg_c |
|  |  |  | GAPD | g3p_c + nad_c + pi_c --> 13dpg_c + h_c + nadh_c |
|  |  |  | ENO | 2pg_c --> h2o_c + pep_c |
|  |  |  | ASPK | asp__L_c + atp_c --> 4pasp_c + adp_c |
|  |  |  | ASAD_r | 4pasp_c + h_c + nadph_c --> aspsa_c + nadp_c + pi_c |
| 5 | 8.390 | 5.208 | PGK_r | 13dpg_c + adp_c --> 3pg_c + atp_c |
|  |  |  | PGM_r | 3pg_c --> 2pg_c |
|  |  |  | GAPD | g3p_c + nad_c + pi_c --> 13dpg_c + h_c + nadh_c |
|  |  |  | ENO | 2pg_c --> h2o_c + pep_c |
|  |  |  | TPI | dhap_c --> g3p_c |
| 6 | 8.919 | 4.767 | PGK_r | 13dpg_c + adp_c --> 3pg_c + atp_c |
|  |  |  | PGM_r | 3pg_c --> 2pg_c |
|  |  |  | GAPD | g3p_c + nad_c + pi_c --> 13dpg_c + h_c + nadh_c |
|  |  |  | ENO | 2pg_c --> h2o_c + pep_c |
|  |  |  | TPI | dhap_c --> g3p_c |
|  |  |  | FBA | fdp_c --> dhap_c + g3p_c |
| 7 | 8.994 | 4.584 | MDH | mal__L_c + nad_c --> h_c + nadh_c + oaa_c |
|  |  |  | FUM | fum_c + h2o_c --> mal__L_c |
| 8 | 17.175 | 1.567 | PGCD | 3pg_c + nad_c --> 3php_c + h_c + nadh_c |
| 9 | 20.079 | 0.522 | ENO_r | h2o_c + pep_c --> 2pg_c |
|  |  |  | PGM | 2pg_c --> 3pg_c |
|  |  |  | PGCD | 3pg_c + nad_c --> 3php_c + h_c + nadh_c |
| 10 | 20.119 | 0.219 | PGCD | 3pg_c + nad_c --> 3php_c + h_c + nadh_c |
|  |  |  | PGK_r | 13dpg_c + adp_c --> 3pg_c + atp_c |
|  |  |  | FLDR2 | 2.0 flxso_c + nadph_c --> 2.0 flxr_c + h_c + nadp_c |
|  |  |  | POR5_r | accoa_c + co2_c + 2.0 flxr_c + h_c --> coa_c + 2.0 flxso_c + pyr_c |
|  |  |  | ENO_r | h2o_c + pep_c --> 2pg_c |
|  |  |  | PPS | atp_c + h2o_c + pyr_c --> amp_c + 2.0 h_c + pep_c + pi_c |
|  |  |  | GAPD | g3p_c + nad_c + pi_c --> 13dpg_c + h_c + nadh_c |
|  |  |  | PGM | 2pg_c --> 3pg_c |
| 11 | 20.661 | 0.069 | PGK_r | 13dpg_c + adp_c --> 3pg_c + atp_c |
|  |  |  | TPI | dhap_c --> g3p_c |
|  |  |  | PGCD | 3pg_c + nad_c --> 3php_c + h_c + nadh_c |
|  |  |  | GAPD | g3p_c + nad_c + pi_c --> 13dpg_c + h_c + nadh_c |
| 12 | 20.833 | -0.482 | PGK_r | 13dpg_c + adp_c --> 3pg_c + atp_c |
|  |  |  | PGCD | 3pg_c + nad_c --> 3php_c + h_c + nadh_c |
|  |  |  | GAPD | g3p_c + nad_c + pi_c --> 13dpg_c + h_c + nadh_c |
|  |  |  | TPI | dhap_c --> g3p_c |
|  |  |  | FBA | fdp_c --> dhap_c + g3p_c |
| 13 | 21.029 | -0.632 | PFL | coa_c + pyr_c --> accoa_c + for_c |
|  |  |  | FLDR2 | 2.0 flxso_c + nadph_c --> 2.0 flxr_c + h_c + nadp_c |
|  |  |  | POR5_r | accoa_c + co2_c + 2.0 flxr_c + h_c --> coa_c + 2.0 flxso_c + pyr_c |

**
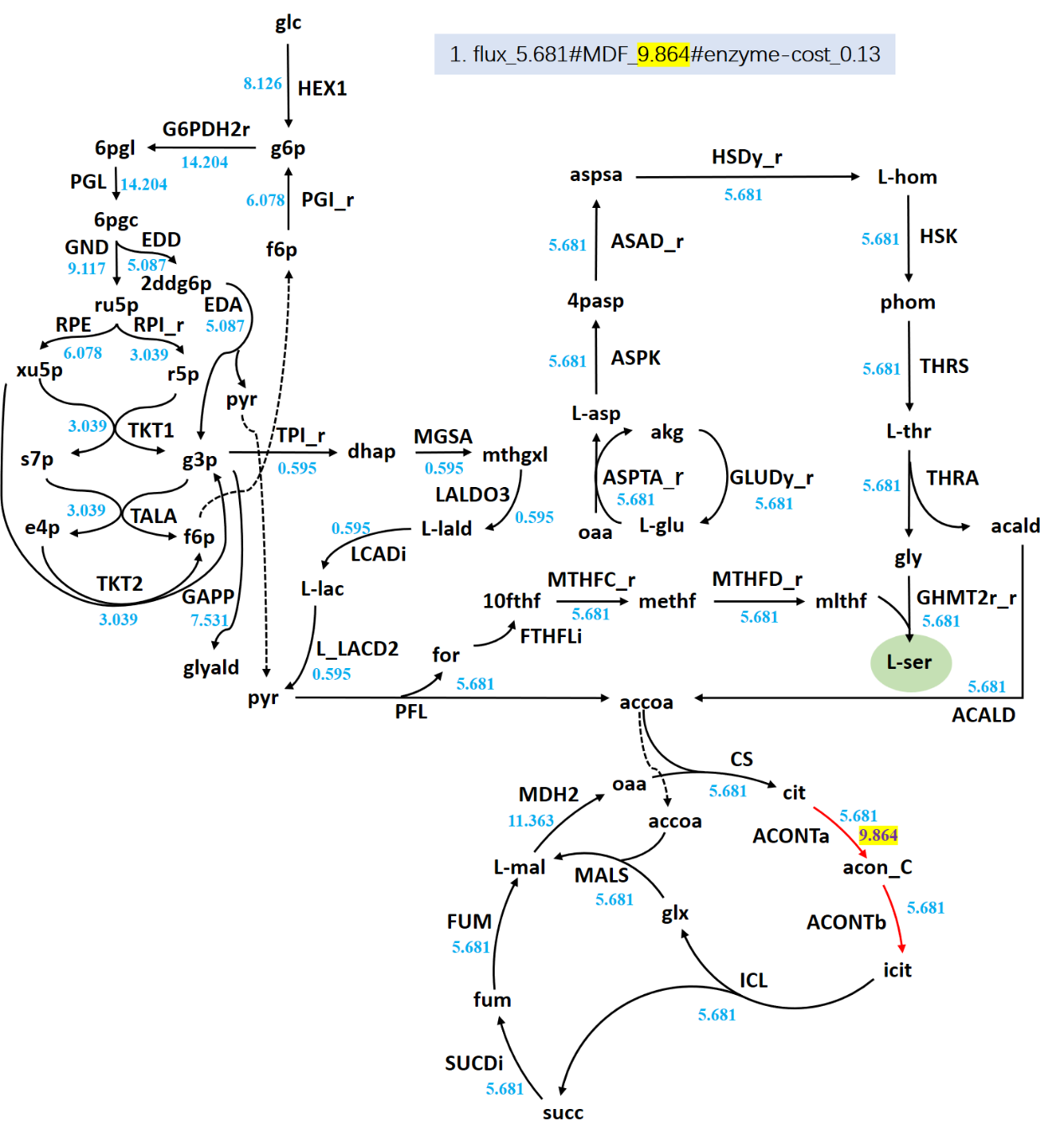

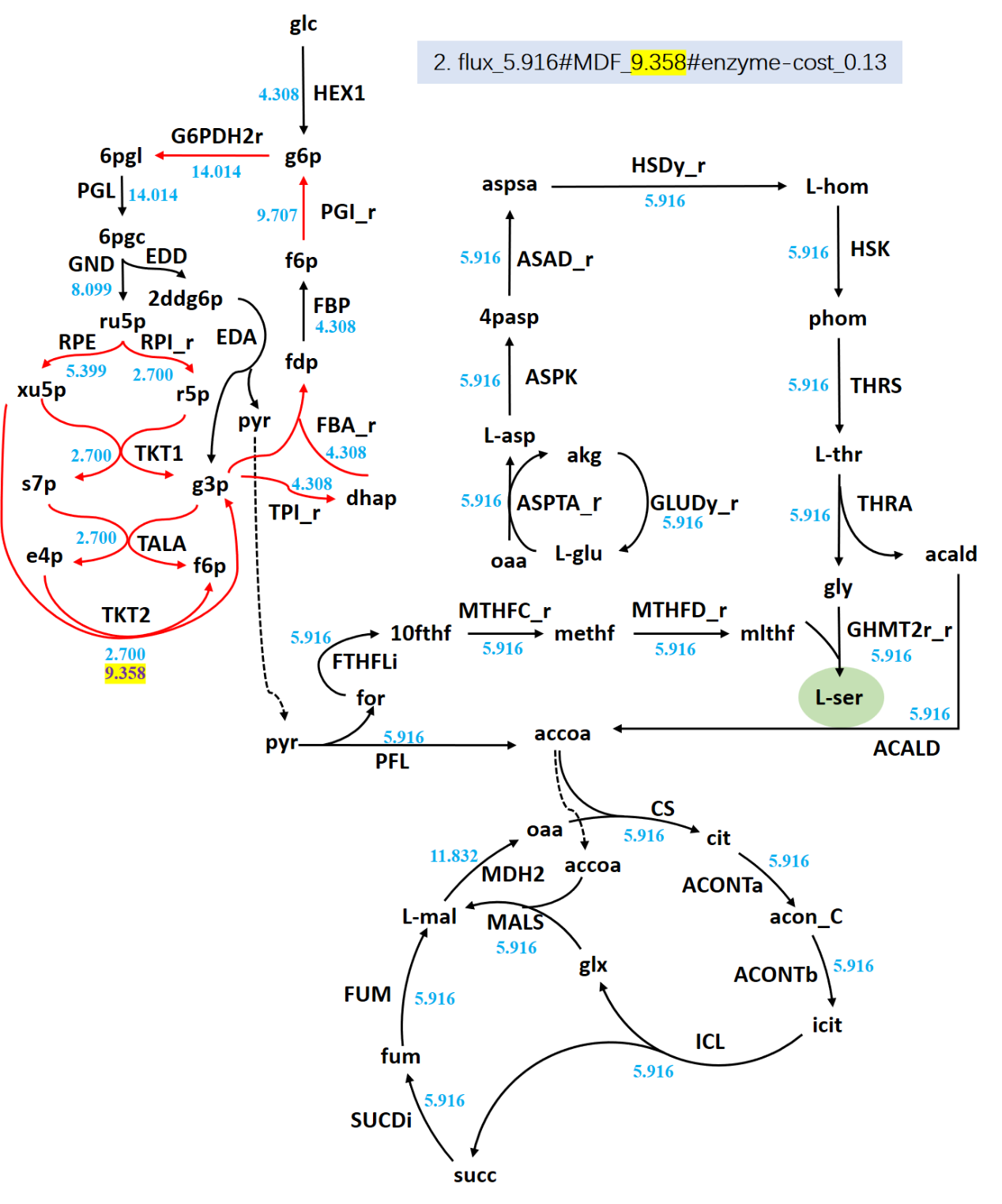

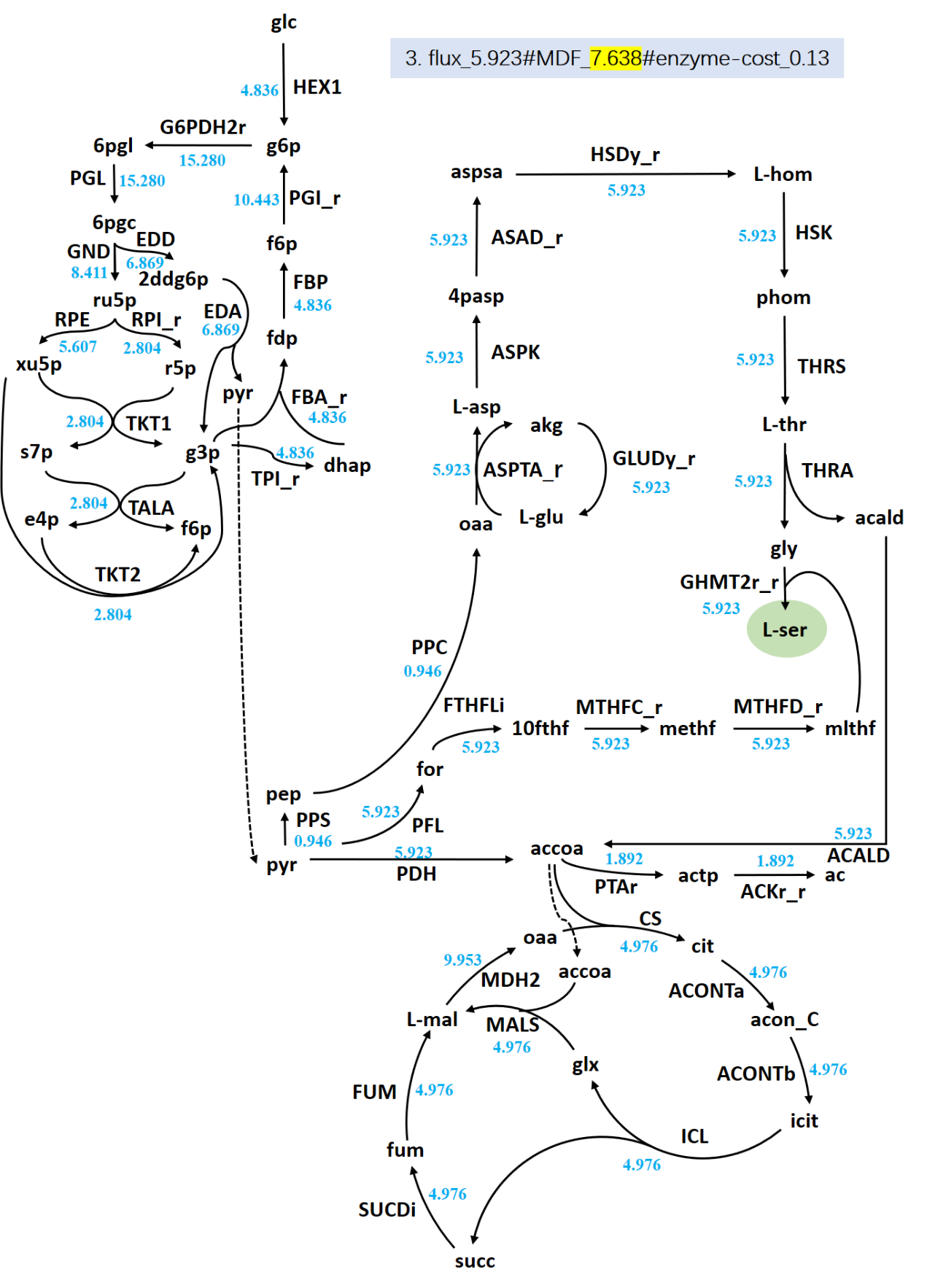

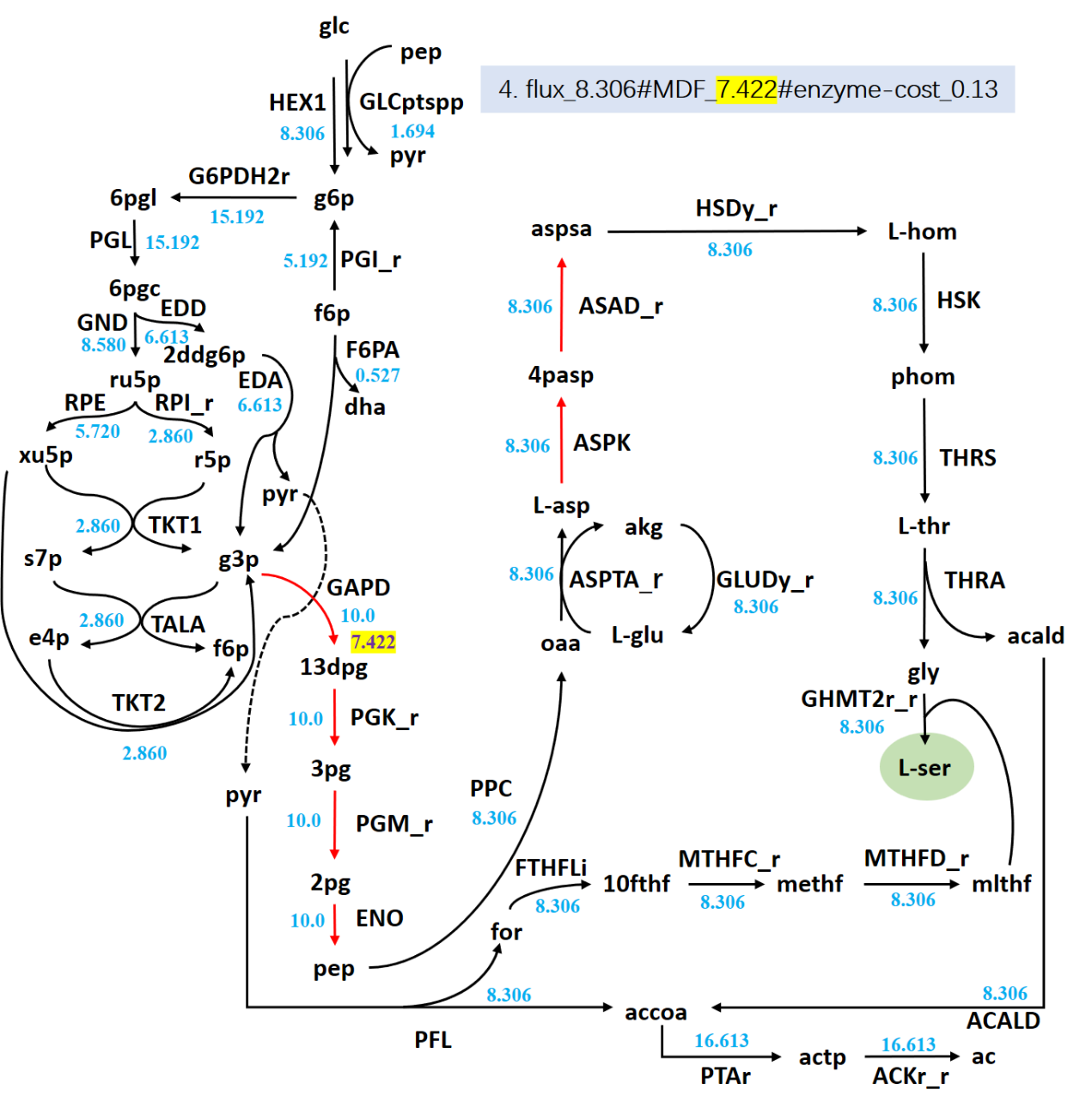

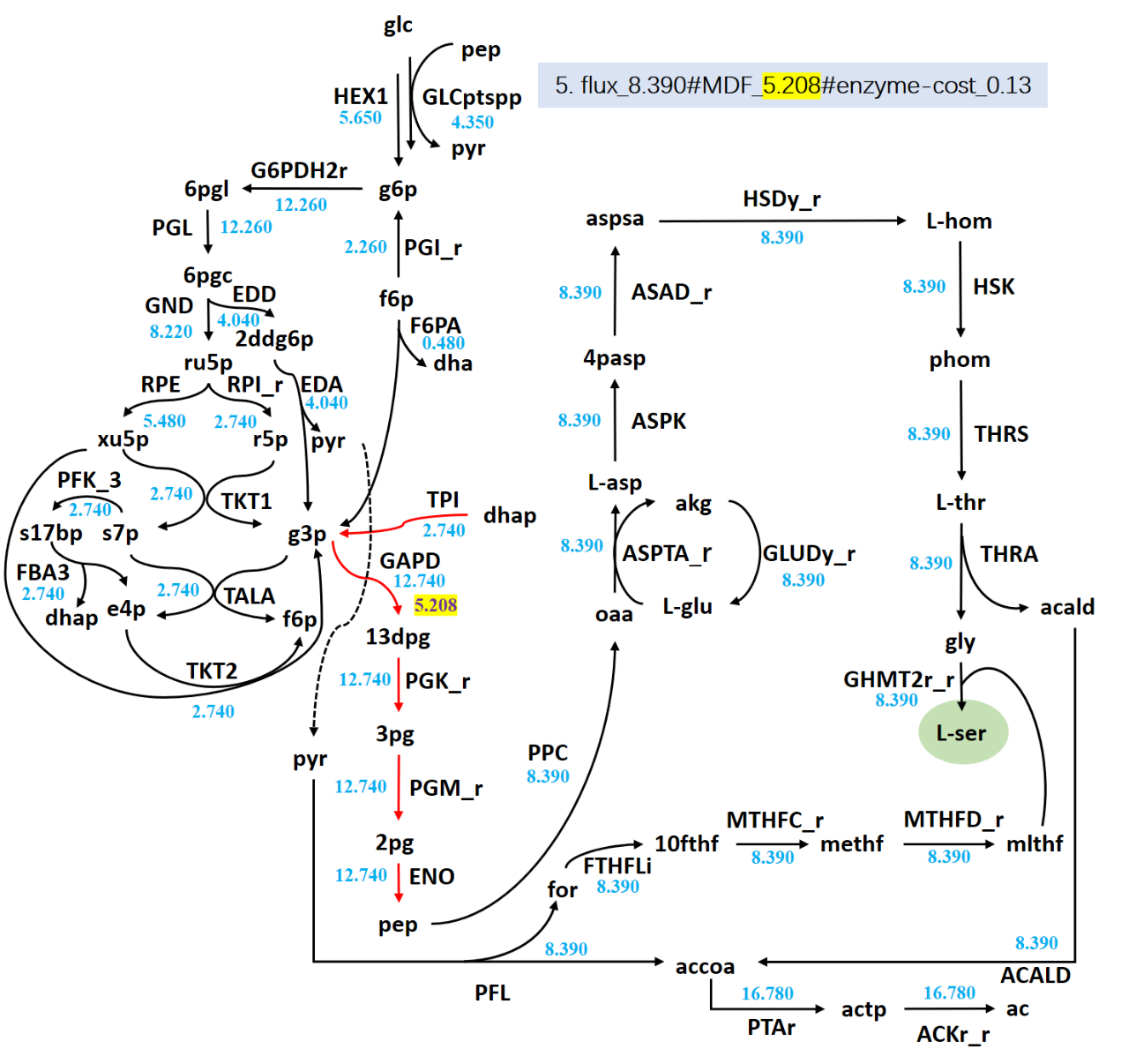

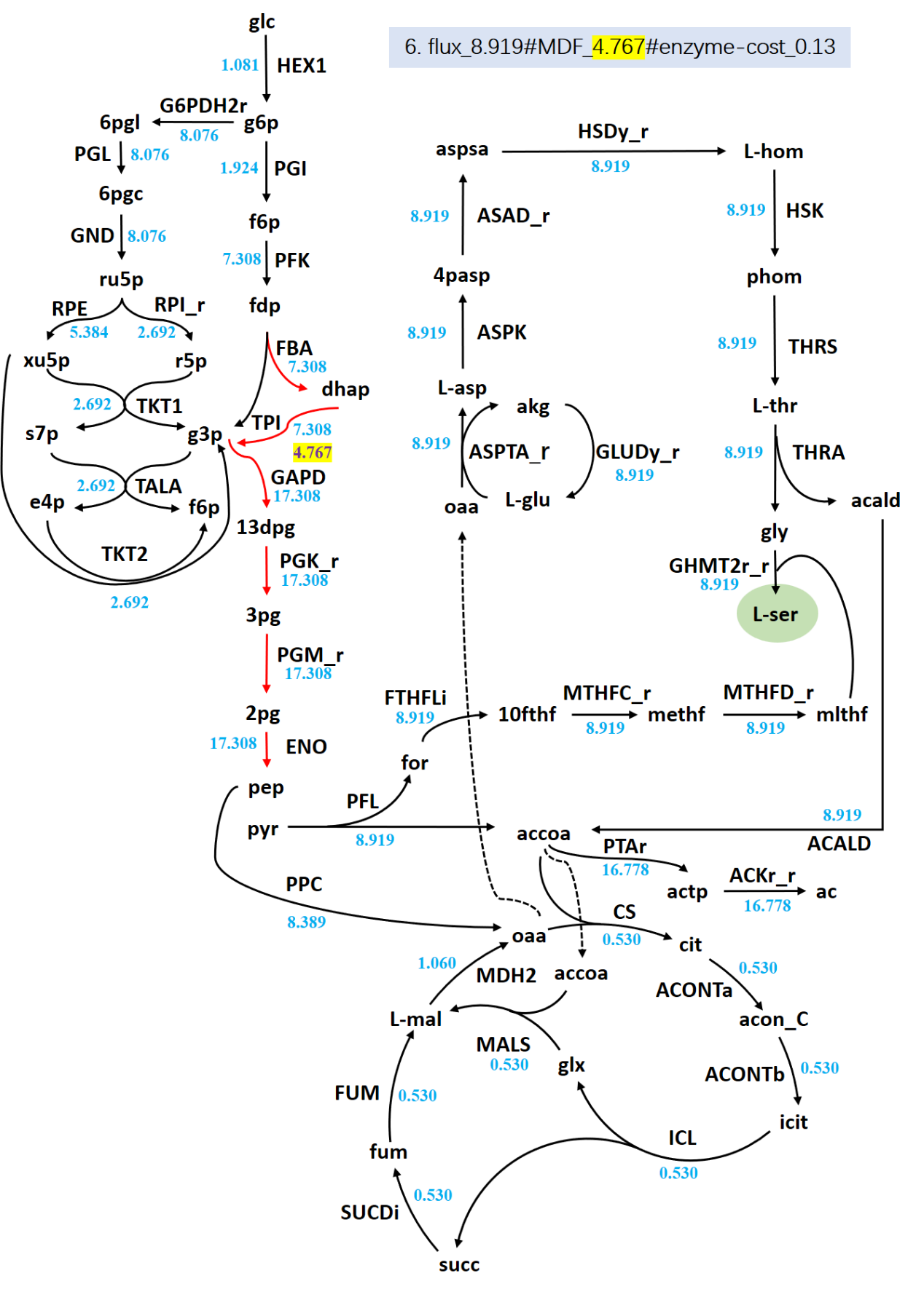

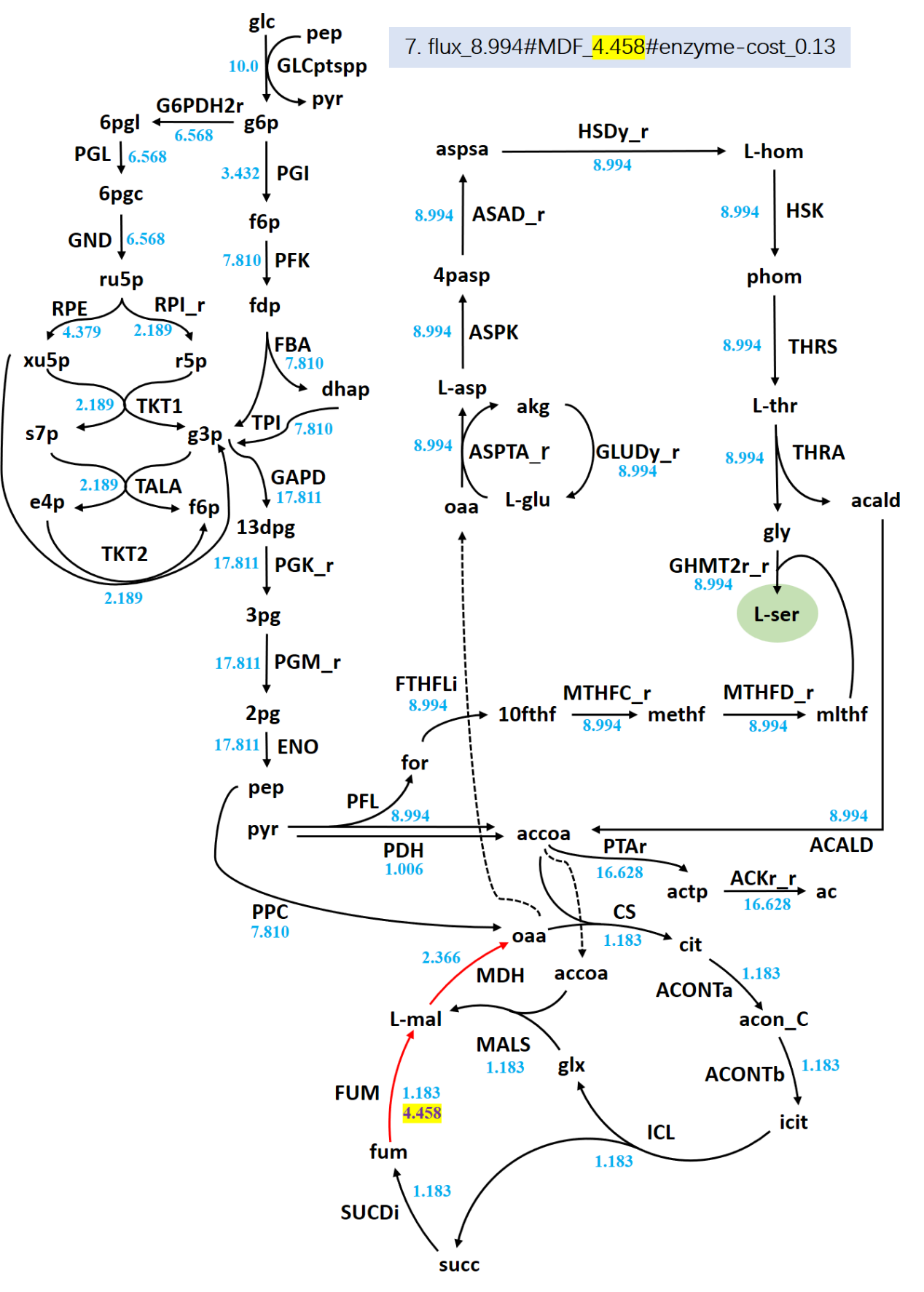

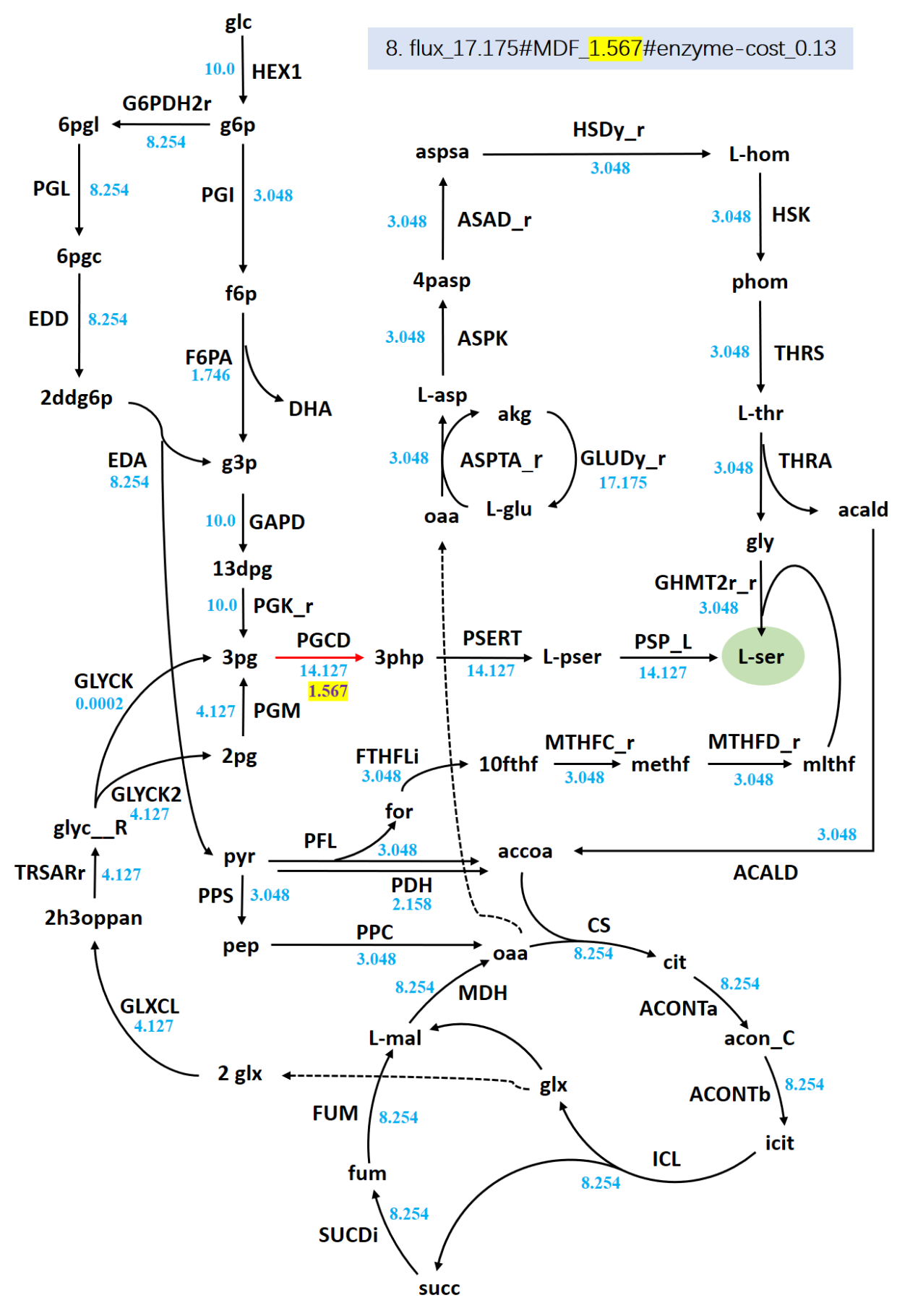

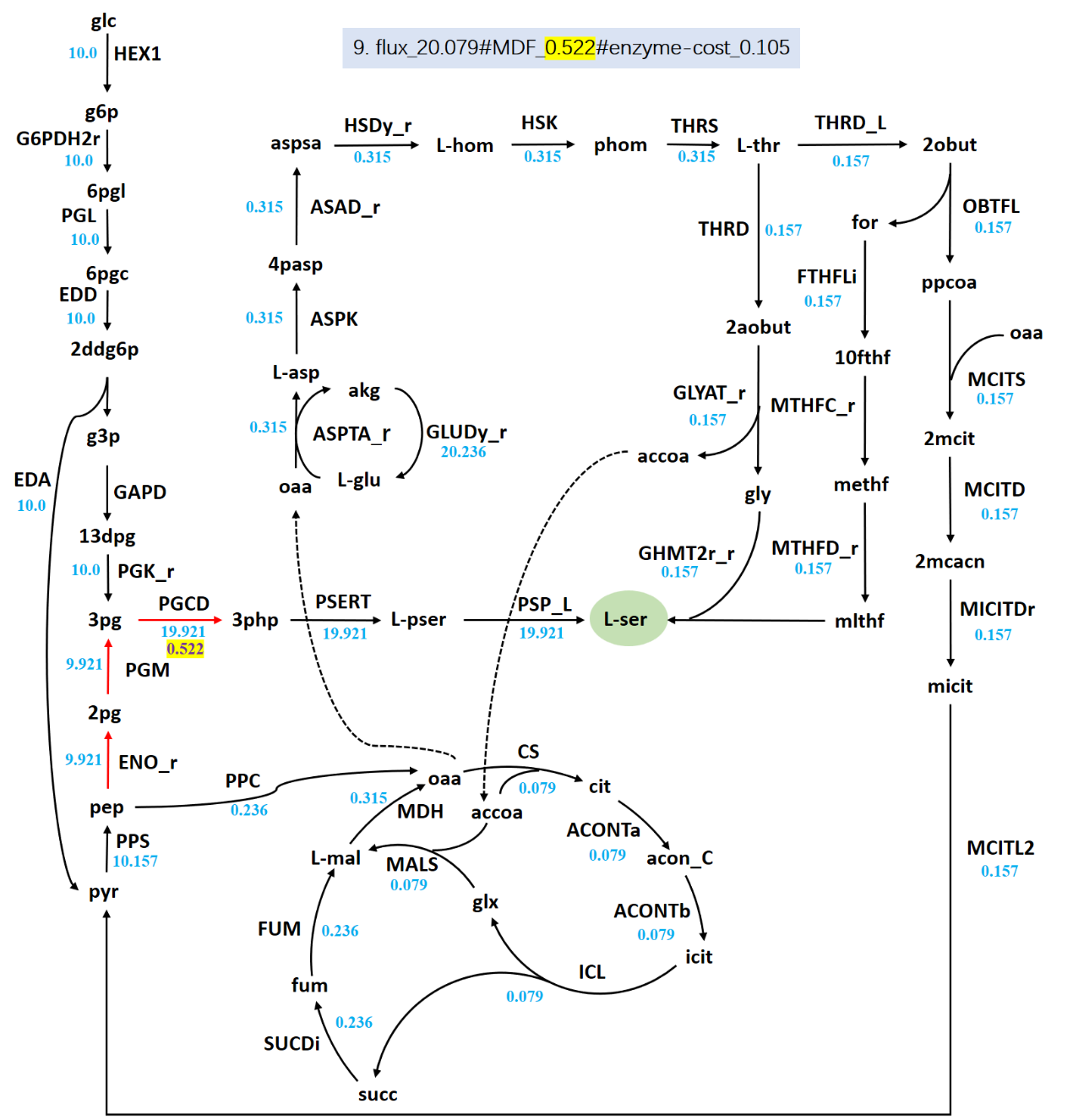

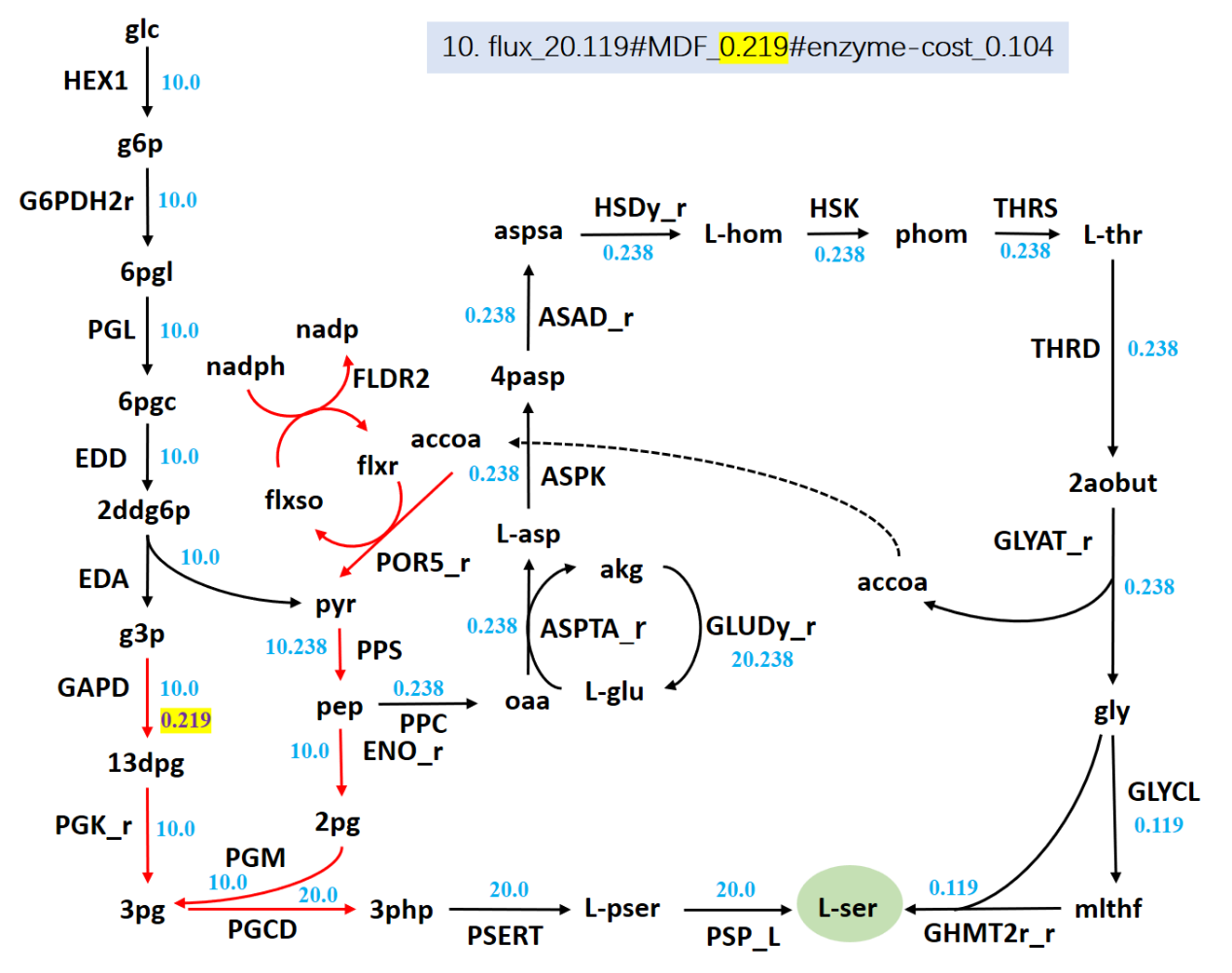
**

**
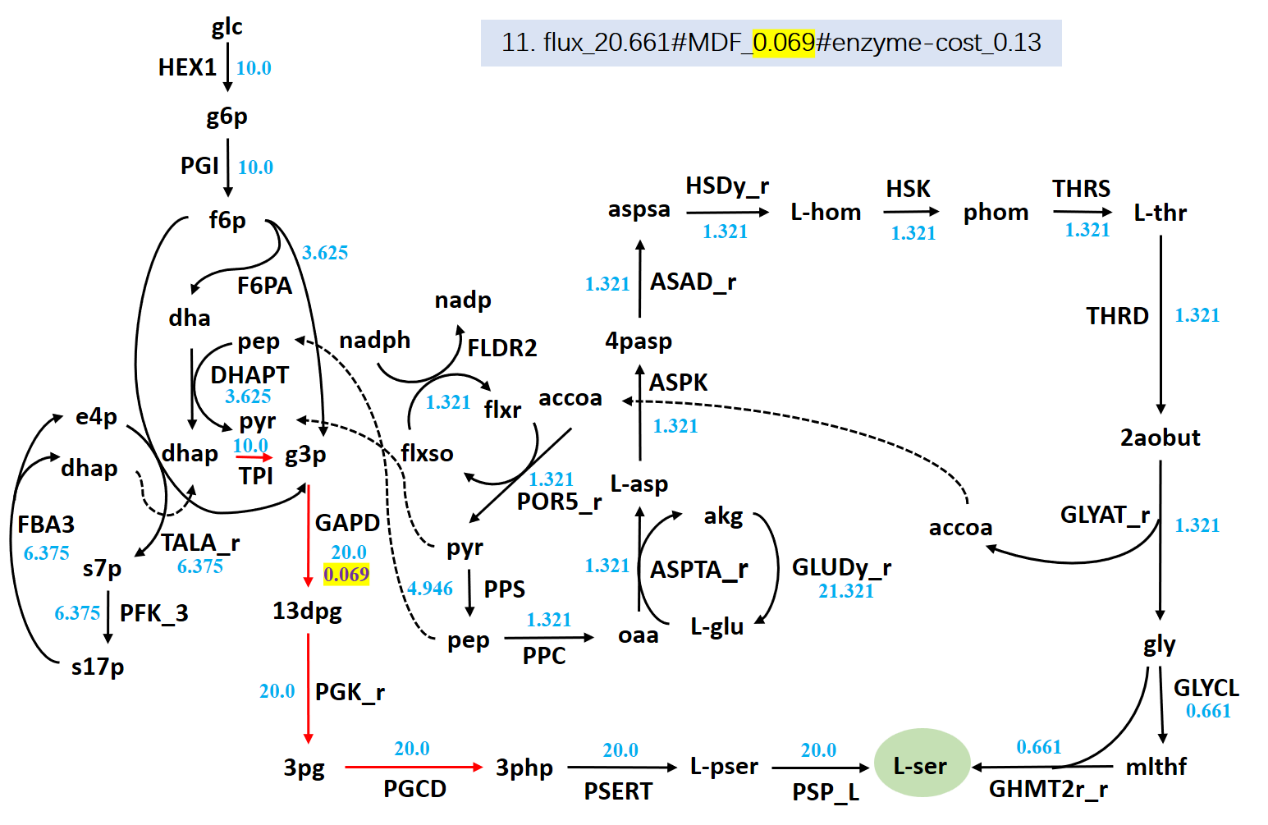
**

**
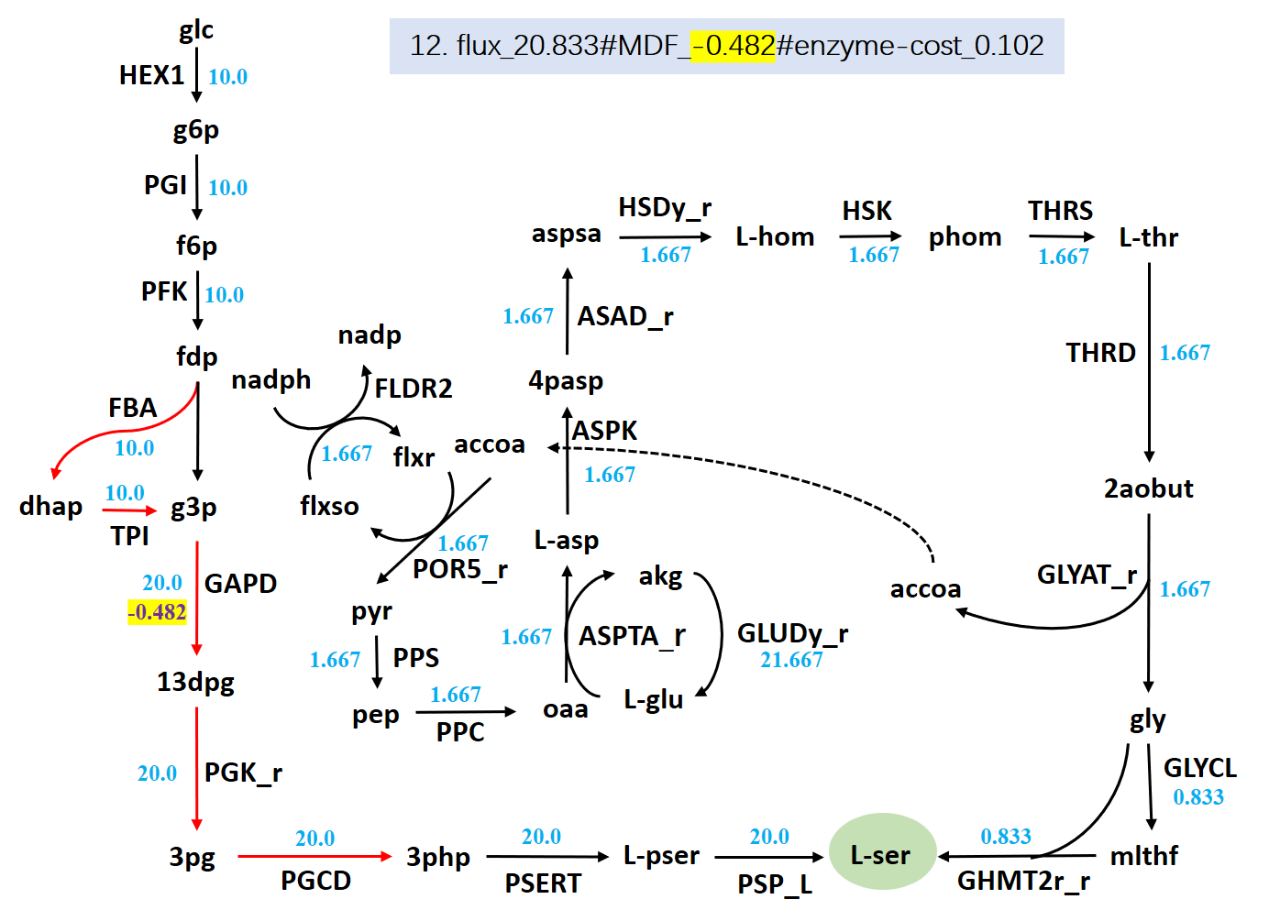
**

**
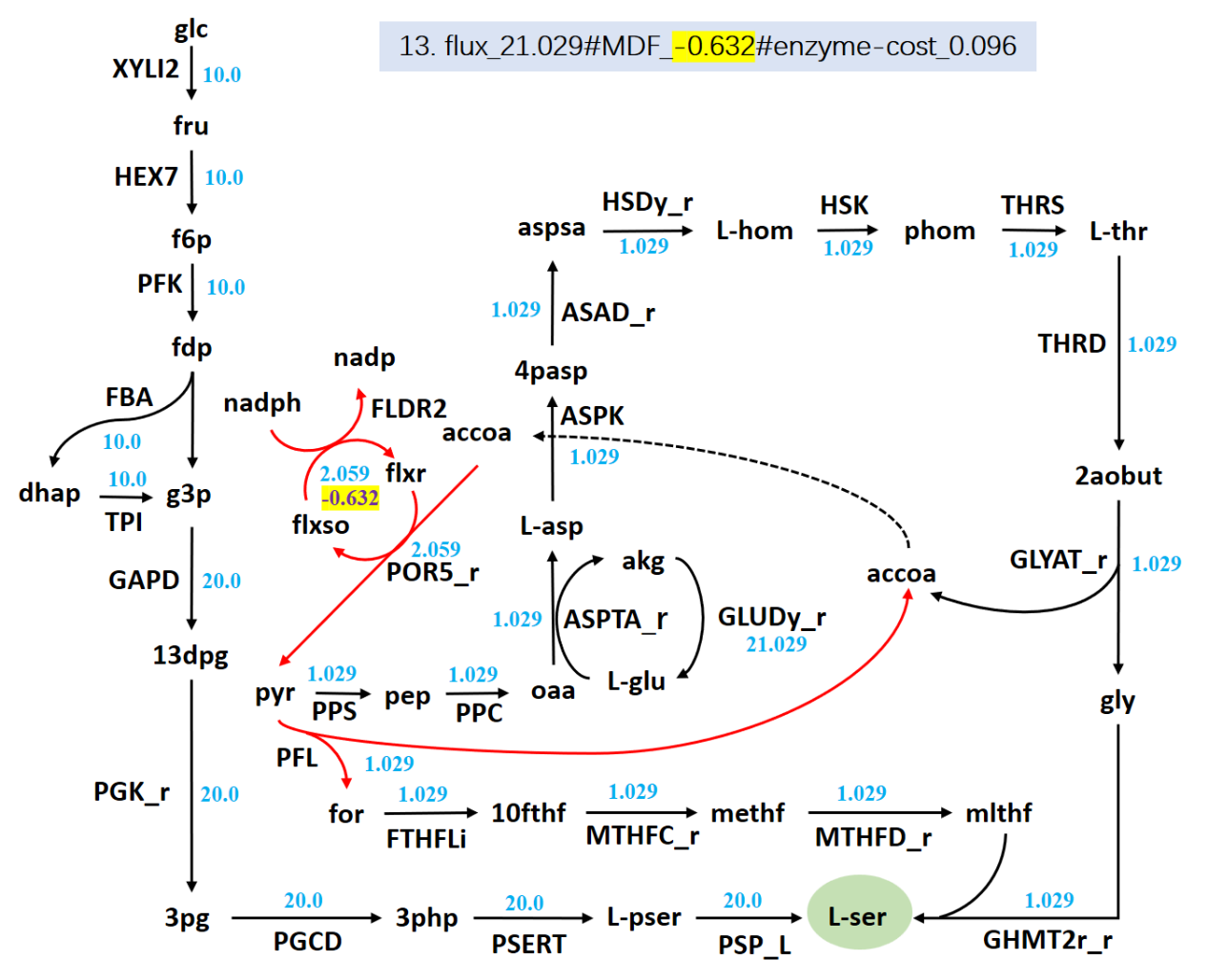
**

**Figure S1.** The 13 l-serine synthesis pathways point-to-point corresponding to Fig. 1 and table S1. Shown are: the thermodynamic bottleneck reaction (red arrow; since their MDF values are all the same, only one value is listed and marked with yellow background.); The unit of the flux value is mmol/gDW/h (blue); and the unit of the maximum thermodynamic driving force is in kJ/mol; the unit of the enzyme cost is g/gDW (it’s up bound is 0.13 g/gDW).


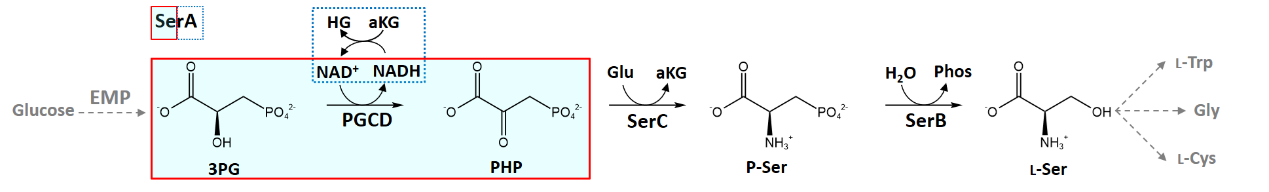


**Figure S2** l-Serine synthesis pathway in *E. coli*. The PGCD reaction was marked by red box; the base-map was referenced the MetaCyc database (<https://metacyc.org/META/NEW-IMAGE?type=PATHWAY&object=SERSYN-PWY&detail-level=3>) [1, 2].

**Table S2** Three distributed bottleneck reactions causing thermodynamics infeasible

| **No.** | **Reaction** | **Reaction equation** | **max*Df_i_*** |
| --- | --- | --- | --- |
|  | **ID** |  | **kJ/mol** |
| 1 | PFL | coa_c + pyr_c --> accoa_c + for_c | 75.86 |
| 2 | FLDR2 | 2.0 flxso_c + nadph_c --> 2.0 flxr_c + h_c + nadp_c | 45.00 |
| 3 | POR5_r | accoa_c + co2_c + 2.0 flxr_c + h_c --> coa_c + 2.0 flxso_c + pyr_c | 68.55 |

**Table S3** Bottleneck reactions of l-serine synthesis pathways after reaction combination.

| **Turning** | **Max flux** | **MDF** | **Bottleneck** | **Reaction equation** |
| --- | --- | --- | --- | --- |
| **point** | **(mmol/gDW/h)** | **(kJ/mol)** | **reaction** |  |
| 1 | 10.00 | 15.77 | PGK_r | 13dpg_c + adp_c --> 3pg_c + atp_c |
|  |  |  | GAPD | g3p_c + nad_c + pi_c --> 13dpg_c + h_c + nadh_c |
| 2 | 13.81 | 9.86 | ACONTa | cit_c --> acon_C_c + h2o_c |
|  |  |  | ACONTb | acon_C_c + h2o_c --> icit_c |
| 3 | 16.06 | 7.64 | ADK1 | amp_c + atp_c --> 2.0 adp_c |
| 4 | 16.57 | 6.64 | TPI | dhap_c --> g3p_c |
|  |  |  | GAPD | g3p_c + nad_c + pi_c --> 13dpg_c + h_c + nadh_c |
| 5 | 18.77 | 5.54 | TPI | dhap_c --> g3p_c |
|  |  |  | GAPD | g3p_c + nad_c + pi_c --> 13dpg_c + h_c + nadh_c |
|  |  |  | FBA | fdp_c --> dhap_c + g3p_c |

**Table S4** The ***∆_r_G_i_′⁰*** information of newly added overall reaction PFPOr

| No. | Reaction ID | Reaction equation | ***∆_r_G_i_′⁰*** | **Notes** |
| --- | --- | --- | --- | --- |
|  |  |  | **kJ/mol** |  |
| **1** | PFL | coa_c + pyr_c --> accoa_c + for_c | -21.2 ± 3 | Original; Shut |
| **2** | FLDR2 | 2.0 flxso_c + nadph_c --> 2.0 flxr_c + h_c + nadp_c | 15.6 ± 14.9 | Original; Shut |
| **3** | POR5_r | accoa_c + co2_c + 2.0 flxr_c + h_c --> coa_c + 2.0 flxso_c + pyr_c | 27.1 ± 16.7 | Original; Shut |
| **4** | PFPOr | co2_c + nadph_c --> for_c + nadp_c | 21.5 ± 22.6 | Newly; added |

**Table S5** The enzyme related parameters of PFPOr

| **No.** | **Reaction** | **Reaction equation** | ***k*_cat_** | **MW** | |
| --- | --- | --- | --- | --- | --- |
|  | **ID** |  | **h^-1^** | **kDa^-1^** | |
| **1** | PFL | coa_c + pyr_c --> accoa_c + for_c | 204410.7 | | 113.56 |
| **2** | FLDR2 | 2.0 flxso_c + nadph_c --> 2.0 flxr_c + h_c + nadp_c | 47286.67 | | 47.45 |
| **3** | POR5_r | accoa_c + co2_c + 2.0 flxr_c + h_c --> coa_c + 2.0 flxso_c + pyr_c | 52980.57 | | 148.52 |
| 4 | PFPOr | co2_c + nadph_c --> for_c + nadp_c | 47286.67 | | 309.53 |

**Table S6** The MDF level of reaction PFPOr.

| **Reaction ID** | **Reaction equation** | **max*Df_i_*** |
| --- | --- | --- |
|  |  | **kJ/mol** |
| PFPOr | co2_c + nadph_c --> for_c + nadp_c | -1.897 |

**Table S7** Bottleneck reactions of anaerobic l-serine synthesis pathways

| **Turning** | **Max flux** | **MDF** | **Bottleneck** | **Reaction equation** |
| --- | --- | --- | --- | --- |
| **point** | **(mmol/gDW/h)** | **(kJ/mol)** | **reaction** |  |
| 1 | 2.069 | 7.422 | PGK_r | 13dpg_c + adp_c --> 3pg_c + atp_c |
|  |  |  | PGM_r | 3pg_c --> 2pg_c |
|  |  |  | GAPD | g3p_c + nad_c + pi_c --> 13dpg_c + h_c + nadh_c |
|  |  |  | ENO | 2pg_c --> h2o_c + pep_c |
|  |  |  | ASPK | asp__L_c + atp_c --> 4pasp_c + adp_c |
|  |  |  | ASAD_r | 4pasp_c + h_c + nadph_c --> aspsa_c + nadp_c + pi_c |
| 2 | 2.963 | 5.208 | PGK_r | 13dpg_c + adp_c --> 3pg_c + atp_c |
|  |  |  | PGM_r | 3pg_c --> 2pg_c |
|  |  |  | GAPD | g3p_c + nad_c + pi_c --> 13dpg_c + h_c + nadh_c |
|  |  |  | ENO | 2pg_c --> h2o_c + pep_c |
|  |  |  | TPI | dhap_c --> g3p_c |
| 3 | 4.706 | -1.897 | PFPOr | co2_c + nadph_c --> for_c + nadp_c |

**
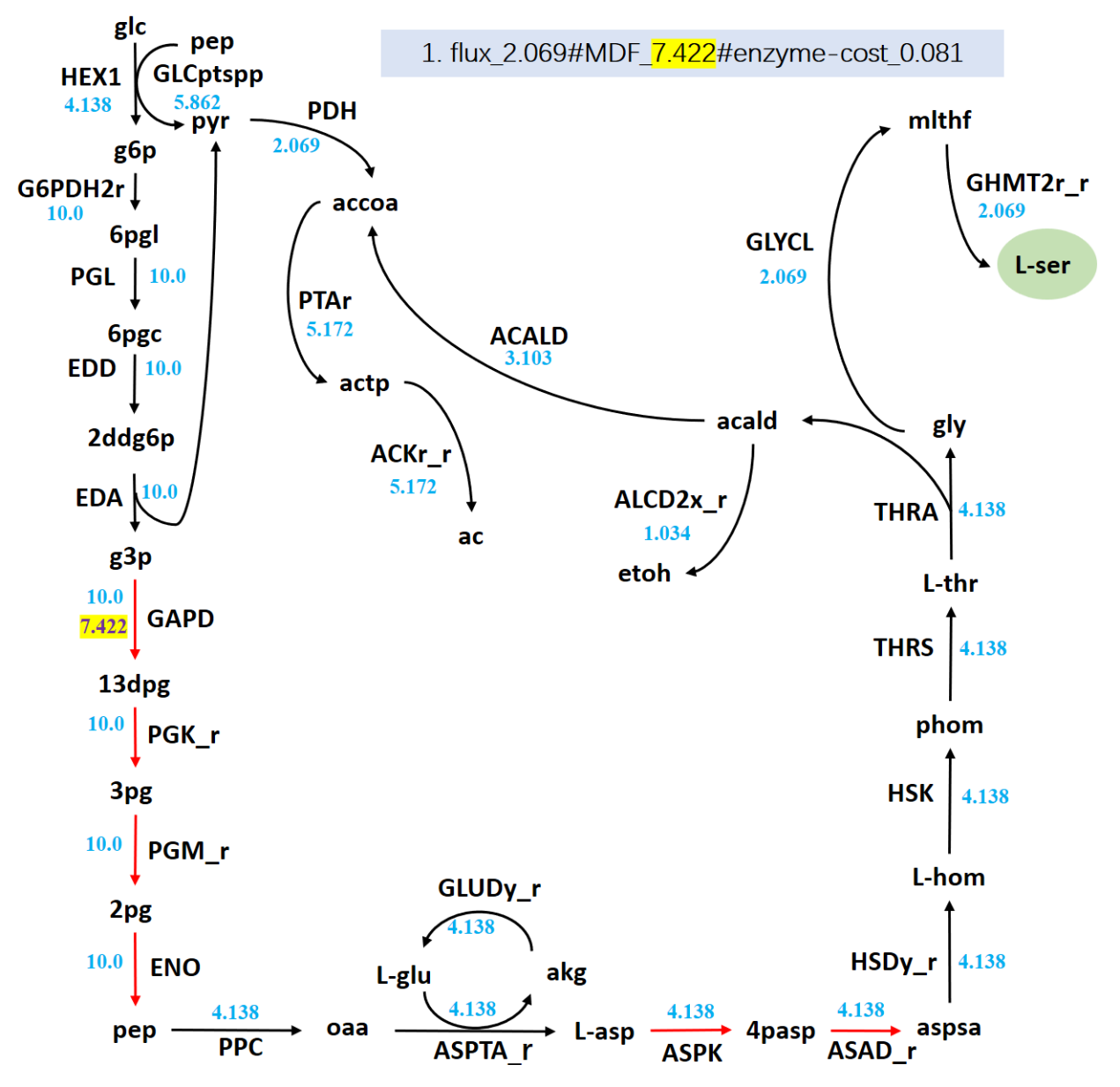

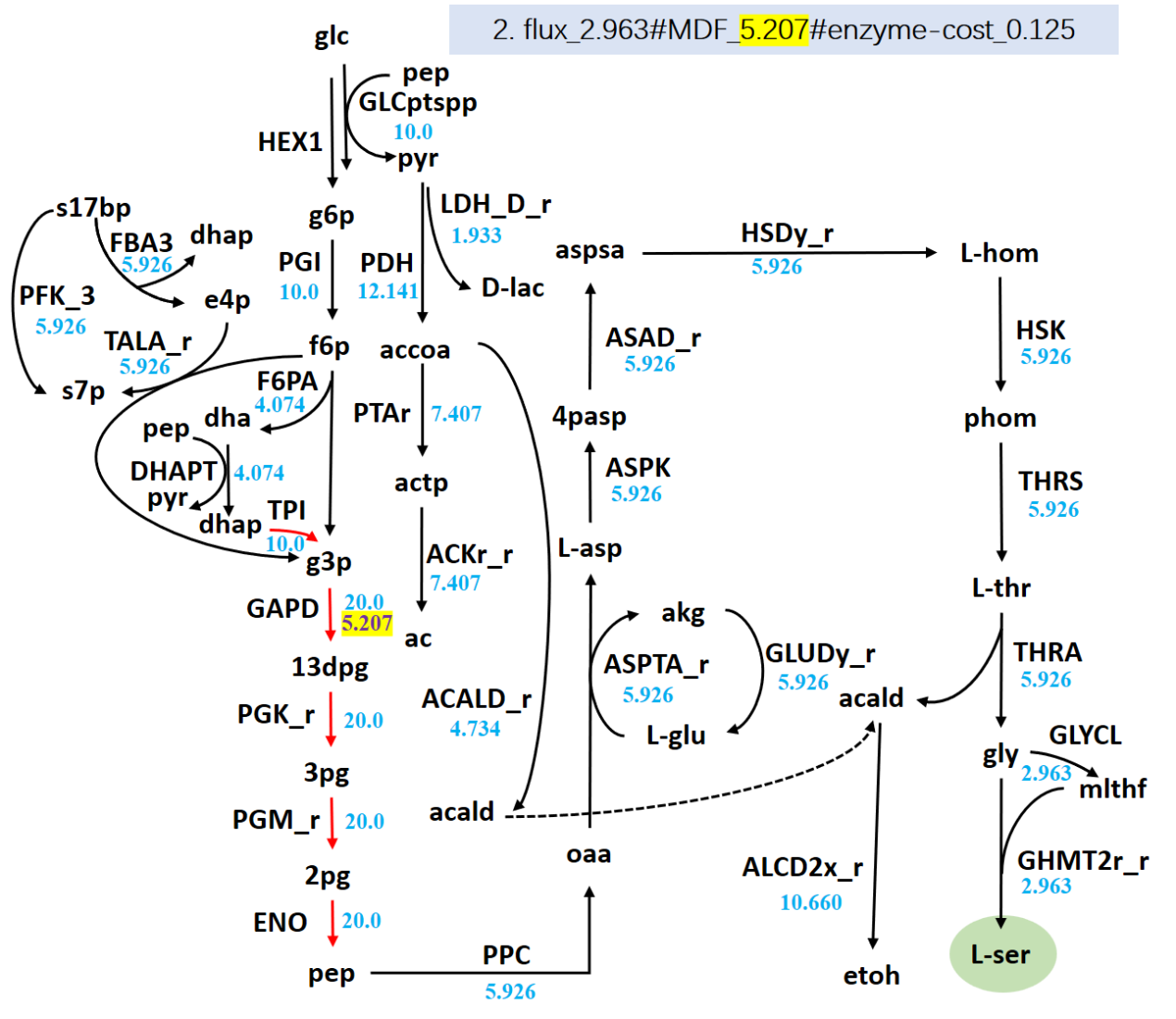
**

**
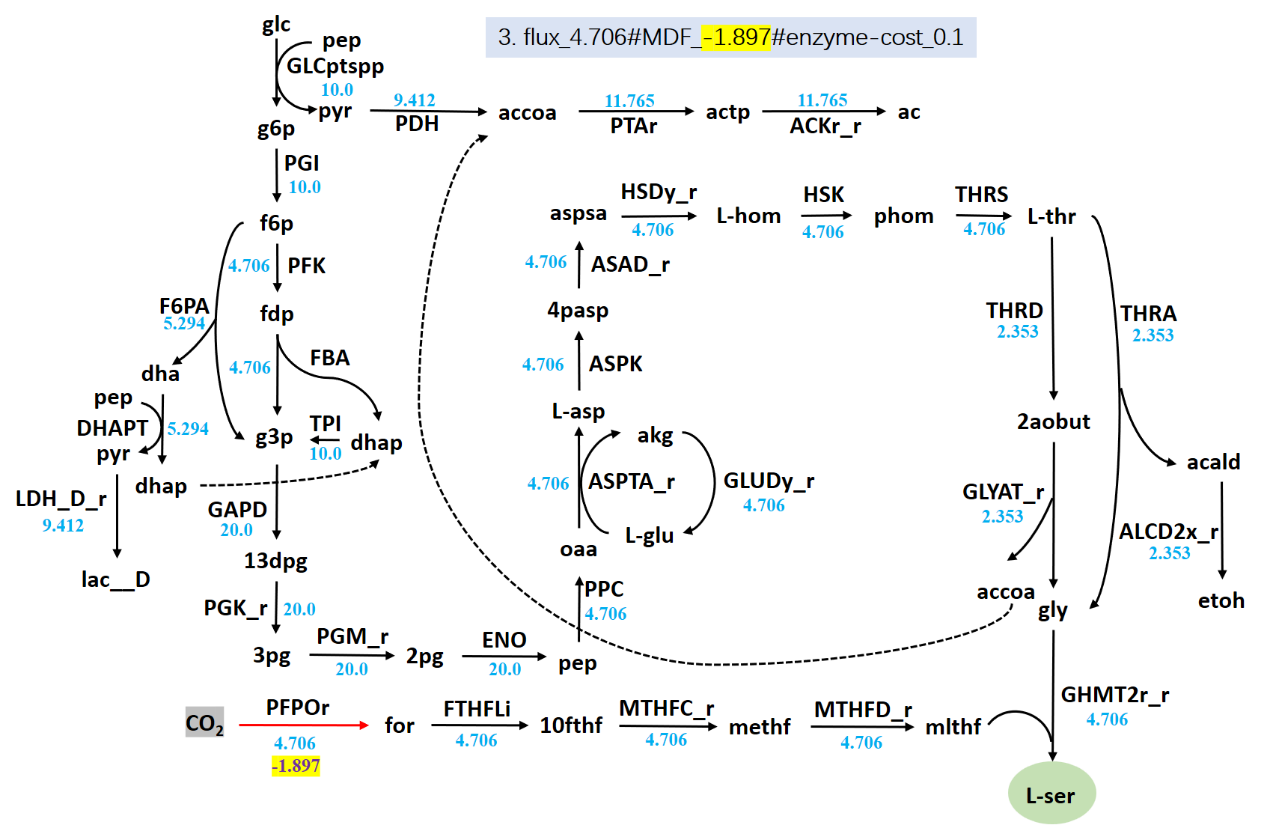
**

**Figure S3** The 3 l-serine synthesis pathways point-to-point corresponding to table S5.**Table S8** The concentration variability of metabolites in reaction PFL, FLDR2 and POR5_r

| **Metabolite** | **min_ln(conc)** | **max_ln(conc)** | **min_conc.**  **μΜ** | **max_conc.**  **μΜ** | **Notes** |
| --- | --- | --- | --- | --- | --- |
| accoa_c | -6.0432 | -3.9120 | 2.3740 | 20.0000 | / |
| pyr_c | -14.5087 | -12.3775 | 0.0005 | 0.0042 | / |
| nadp_c | -14.5087 | -6.2146 | 0.0005 | 2.0000 | / |
| nadph_c | -12.2061 | -3.9120 | 0.0050 | 20.0000 | / |
| for_c | -14.5087 | -14.5087 | 0.0005 | 0.0005 | lower bound |
| flxr_c | -14.5087 | -5.6625 | 0.0005 | 3.4737 | / |
| co2_c | -9.2103 | -9.2103 | 0.1000 | 0.1000 | up bound |
| flxso_c | -12.7581 | -3.9120 | 0.0029 | 20.0000 | / |
| coa_c | -14.5087 | -12.3775 | 0.0005 | 0.0042 | / |

**Table S9**  The concentration variability of metabolites in overall reaction PFPOr

| **Metabolite** | **min_ln(conc)** | **max_ln(conc)** | **min_conc.**  **μΜ** | **max_conc.**  **μΜ** | **Notes** |
| --- | --- | --- | --- | --- | --- |
| co2_c | -9.2103 | -9.2103 | 0.1000 | 0.1000 | up bound |
| nadph_c | -12.2061 | -3.9120 | 0.0050 | 20.0000 | / |
| nadp_c | -14.5087 | -6.2146 | 0.0005 | 2.0000 | / |
| for_c | -14.5087 | -14.5087 | 0.0005 | 0.0005 | lower bound |

**Table S10** Thermodynamic bottleneck reaction(s) in the L-Trp synthesis pathways predicted by the uncorrected EcoETM

| **Turning** | **Max flux** | **MDF** | **Bottleneck** | **Reaction equation** |
| --- | --- | --- | --- | --- |
| **point** | **(mmol/gDW/h)** | **(kJ/mol)** | **reaction** |  |
| 1 | 1.74 | 9.86 | ACONTa | cit_c --> acon_C_c + h2o_c |
|  |  |  | ACONTb | acon_C_c + h2o_c --> icit_c |
| 2 | 1.78 | 9.82 | TKT2_r | f6p_c + g3p_c --> e4p_c + xu5p__D_c |
|  |  |  | RPI_r | ru5p__D_c --> r5p_c |
|  |  |  | TALA | g3p_c + s7p_c --> e4p_c + f6p_c |
|  |  |  | TKT1 | r5p_c + xu5p__D_c --> g3p_c + s7p_c |
|  |  |  | EDA | 2ddg6p_c --> g3p_c + pyr_c |
|  |  |  | TRPS3 | 3ig3p_c --> g3p_c + indole_c |
| 3 | 1.79 | 9.75 | TPI_r | g3p_c --> dhap_c |
|  |  |  | FBA_r | dhap_c + g3p_c --> fdp_c |
|  |  |  | TRPS3 | 3ig3p_c --> g3p_c + indole_c |
|  |  |  | EDA | 2ddg6p_c --> g3p_c + pyr_c |
|  |  |  | TRPAS2_r | indole_c + nh4_c + pyr_c --> h2o_c + trp__L_c |
| 4 | 1.91 | 9.39 | PRPPS | atp_c + r5p_c --> amp_c + h_c + prpp_c |
|  |  |  | RPI_r | ru5p__D_c --> r5p_c |
|  |  |  | RPE_r | xu5p__D_c --> ru5p__D_c |
|  |  |  | TKT2_r | f6p_c + g3p_c --> e4p_c + xu5p__D_c |
|  |  |  | TRPS3 | 3ig3p_c --> g3p_c + indole_c |
|  |  |  | EDA | 2ddg6p_c --> g3p_c + pyr_c |
|  |  |  | TRPAS2_r | indole_c + nh4_c + pyr_c --> h2o_c + trp__L_c |
| 5 | 2.17 | 8.78 | RPI_r | ru5p__D_c --> r5p_c |
|  |  |  | PPM_r | r5p_c --> r1p_c |
| 6 | 2.87 | 7.64 | ADK1 | amp_c + atp_c <=> 2.0 adp_c |
| 7 | 3.66 | 7.16 | PGK_r | 13dpg_c + adp_c --> 3pg_c + atp_c |
|  |  |  | TRPS3 | 3ig3p_c --> g3p_c + indole_c |
|  |  |  | PGM_r | 3pg_c --> 2pg_c |
|  |  |  | TRPAS2_r | indole_c + nh4_c + pyr_c --> h2o_c + trp__L_c |
|  |  |  | GAPD | g3p_c + nad_c + pi_c --> 13dpg_c + h_c + nadh_c |
|  |  |  | ENO | 2pg_c --> h2o_c + pep_c |
| 8 | 3.82 | 6.28 | PGK_r | 13dpg_c + adp_c --> 3pg_c + atp_c |
|  |  |  | PGM_r | 3pg_c --> 2pg_c |
|  |  |  | GAPD | g3p_c + nad_c + pi_c --> 13dpg_c + h_c + nadh_c |
|  |  |  | ENO | 2pg_c --> h2o_c + pep_c |
|  |  |  | TRPS3 | 3ig3p_c --> g3p_c + indole_c |
|  |  |  | TRPAS2_r | indole_c + nh4_c + pyr_c --> h2o_c + trp__L_c |
| 9 | 3.85 | 5.21 | PGK_r | 13dpg_c + adp_c --> 3pg_c + atp_c |
|  |  |  | PGM_r | 3pg_c --> 2pg_c |
|  |  |  | GAPD | g3p_c + nad_c + pi_c --> 13dpg_c + h_c + nadh_c |
|  |  |  | ENO | 2pg_c --> h2o_c + pep_c |
|  |  |  | TPI | dhap_c --> g3p_c |
| 10 | 4.13 | 4.77 | PGK_r | 13dpg_c + adp_c --> 3pg_c + atp_c |
|  |  |  | PGM_r | 3pg_c --> 2pg_c |
|  |  |  | GAPD | g3p_c + nad_c + pi_c --> 13dpg_c + h_c + nadh_c |
|  |  |  | ENO | 2pg_c --> h2o_c + pep_c |
|  |  |  | TPI | dhap_c --> g3p_c |
|  |  |  | FBA | fdp_c --> dhap_c + g3p_c |


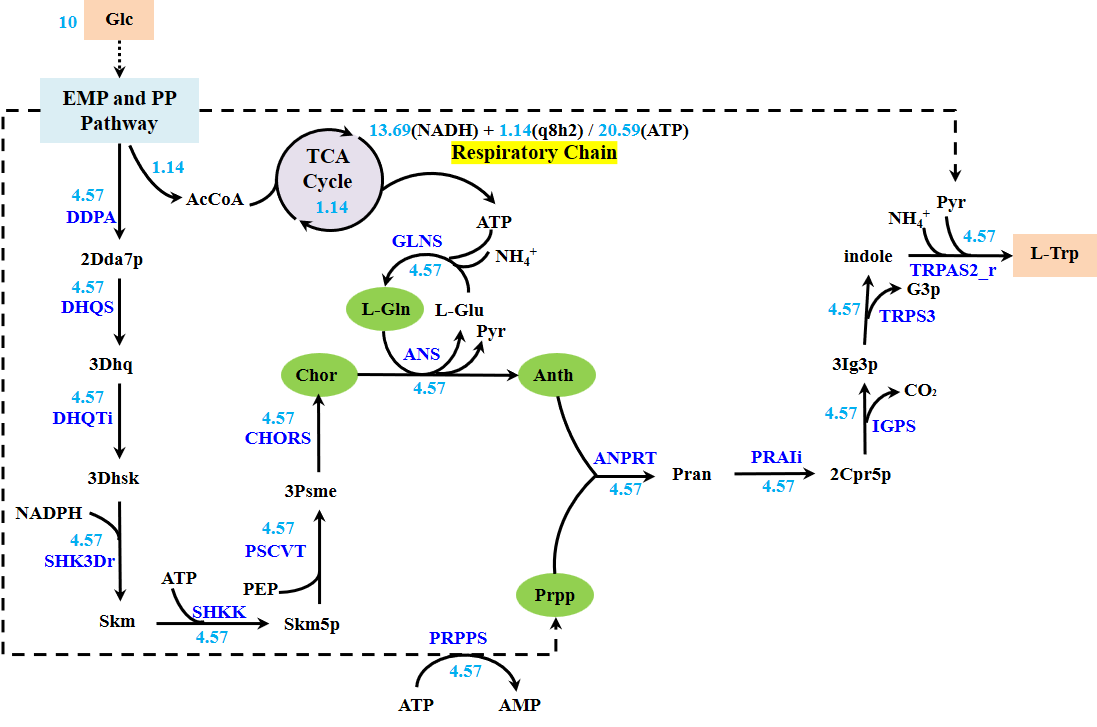


**Figure S4** l-Trp synthesis pathway in *E. coli* predicted by *i*ML1515. The unit of the flux value is mmol/gDW/h (blue).


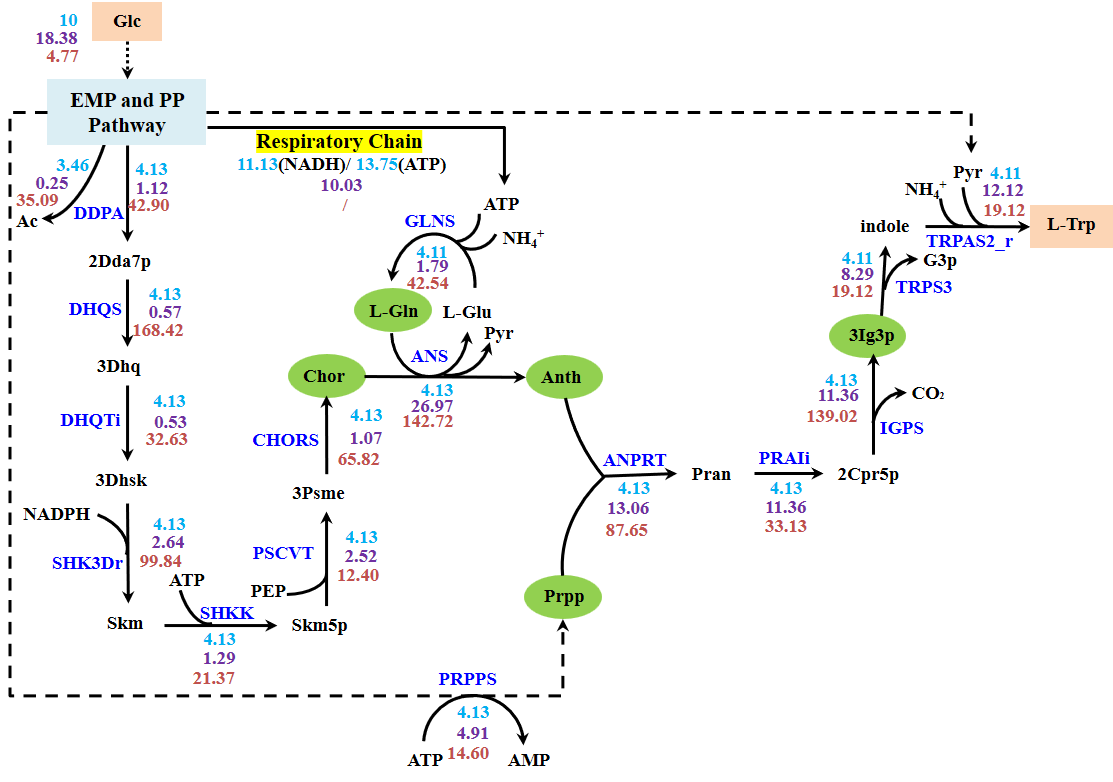


**Figure S5** l-Trp synthesis pathway in *E. coli* predicted by uncorrected EcoETM. The unit of the flux value is mmol/gDW/h (blue, on top), the unit of the enzyme cost is mg/gDW (purple, in the middle), and the unit of the maximal thermodynamic driving force is kJ/mol (orange, at the bottom).

**Table S11** Thermodynamic bottleneck reaction(s) in the l-Trp synthesis processes predicted by the corrected EcoETM

| **Turning** | **Max flux** | **MDF** | **Bottleneck** | **Reaction equation** |
| --- | --- | --- | --- | --- |
| **point** | **(mmol/gDW/h)** | **(kJ/mol)** | **reaction(s)** |  |
| 1 | 1.29 | 17.42 | PRPPS | atp_c + r5p_c --> amp_c + h_c + prpp_c |
|  |  |  | RPI_r | ru5p__D_c --> r5p_c |
| 2 | 1.30 | 17.21 | RPI_r | ru5p__D_c --> r5p_c |
|  |  |  | TPI_r | g3p_c --> dhap_c |
|  |  |  | RPE | ru5p__D_c --> xu5p__D_c |
|  |  |  | PFK_3 | atp_c + s7p_c --> adp_c + h_c + s17bp_c |
|  |  |  | TKT1 | r5p_c + xu5p__D_c --> g3p_c + s7p_c |
| 3 | 1.38 | 16.76 | RPI_r | ru5p__D_c --> r5p_c |
|  |  |  | TKT2_r | f6p_c + g3p_c --> e4p_c + xu5p__D_c |
|  |  |  | TKT1 | r5p_c + xu5p__D_c --> g3p_c + s7p_c |
|  |  |  | PFK_3 | atp_c + s7p_c --> adp_c + h_c + s17bp_c |
| 4 | 1.40 | 16.30 | PGL | 6pgl_c + h2o_c --> 6pgc_c + h_c |
|  |  |  | G6PDH2r | g6p_c + nadp_c --> 6pgl_c + h_c + nadph_c |
|  |  |  | GND | 6pgc_c + nadp_c --> co2_c + nadph_c + ru5p__D_c |
|  |  |  | TKT2_r | f6p_c + g3p_c --> e4p_c + xu5p__D_c |
|  |  |  | RPI_r | ru5p__D_c --> r5p_c |
|  |  |  | TKT1 | r5p_c + xu5p__D_c --> g3p_c + s7p_c |
| 5 | 1.42 | 14.87 | ASPK | asp__L_c + atp_c <=> 4pasp_c + adp_c |
| 6 | 1.90 | 14.71 | HEX7 | atp_c + fru_c --> adp_c + f6p_c + h_c |
|  |  |  | TKT2_r | f6p_c + g3p_c --> e4p_c + xu5p__D_c |
|  |  |  | RPI_r | ru5p__D_c --> r5p_c |
|  |  |  | TKT1 | r5p_c + xu5p__D_c --> g3p_c + s7p_c |
|  |  |  | XYLI2 | glc__D_c --> fru_c |
| 7 | 1.92 | 14.17 | PGK_r | 13dpg_c + adp_c --> 3pg_c + atp_c |
|  |  |  | FBP | fdp_c + h2o_c --> f6p_c + pi_c |
|  |  |  | FBA_r | dhap_c + g3p_c --> fdp_c |
|  |  |  | RPI_r | ru5p__D_c --> r5p_c |
|  |  |  | TKT1 | r5p_c + xu5p__D_c --> g3p_c + s7p_c |
|  |  |  | FBA3 | s17bp_c --> dhap_c + e4p_c |
|  |  |  | PFK_3 | atp_c + s7p_c --> adp_c + h_c + s17bp_c |
|  |  |  | GAPD | 2pg_c --> h2o_c + pep_c |
|  |  |  | TKT2_r | f6p_c + g3p_c --> e4p_c + xu5p__D_c |
| 8 | 1.97 | 12.75 | TALA | g3p_c + s7p_c --> e4p_c + f6p_c |
|  |  |  | TKT2_r | f6p_c + g3p_c --> e4p_c + xu5p__D_c |
|  |  |  | RPI_r | ru5p__D_c --> r5p_c |
|  |  |  | TKT1 | r5p_c + xu5p__D_c --> g3p_c + s7p_c |
| 9 | 2.03 | 11.21 | TALA | g3p_c + s7p_c --> e4p_c + f6p_c |
|  |  |  | PGI | g6p_c --> f6p_c |
|  |  |  | TKT2_r | f6p_c + g3p_c --> e4p_c + xu5p__D_c |
|  |  |  | RPI_r | ru5p__D_c --> r5p_c |
|  |  |  | TKT1 | r5p_c + xu5p__D_c --> g3p_c + s7p_c |
|  |  |  | F6PA | f6p_c --> dha_c + g3p_c |
| 10 | 2.06 | 9.23 | PGI | g6p_c --> f6p_c |
|  |  |  | TKT2_r | f6p_c + g3p_c --> e4p_c + xu5p__D_c |
|  |  |  | RPI_r | ru5p__D_c --> r5p_c |
|  |  |  | RPE_r | xu5p__D_c --> ru5p__D_c |
|  |  |  | PRPPS | atp_c + r5p_c --> amp_c + h_c + prpp_c |
| 11 | 2.07 | 8.92 | PRPPS | atp_c + r5p_c --> amp_c + h_c + prpp_c |
|  |  |  | PGI | g6p_c --> f6p_c |
|  |  |  | TKT2_r | f6p_c + g3p_c --> e4p_c + xu5p__D_c |
|  |  |  | RPI_r | ru5p__D_c --> r5p_c |
|  |  |  | F6PA | f6p_c --> dha_c + g3p_c |
|  |  |  | RPE_r | xu5p__D_c --> ru5p__D_c |
| 12 | 2.34 | 8.78 | RPI_r | ru5p__D_c --> r5p_c |
|  |  |  | PPM_r | r5p_c --> r1p_c |
| 13 | 2.87 | 7.88 | PGK_r | 13dpg_c + adp_c --> 3pg_c + atp_c |
|  |  |  | PGM_r | 3pg_c --> 2pg_c |
|  |  |  | ENO | g3p_c + nad_c + pi_c --> 13dpg_c + h_c + nadh_c |
|  |  |  | GAPD | 2pg_c --> h2o_c + pep_c |
| 14 | 3.10 | 7.64 | ADK1 | amp_c + atp_c <=> 2.0 adp_c |
| 15 | 3.12 | 5.21 | PGK_r | 13dpg_c + adp_c --> 3pg_c + atp_c |
|  |  |  | PGM_r | 3pg_c --> 2pg_c |
|  |  |  | GAPD | g3p_c + nad_c + pi_c --> 13dpg_c + h_c + nadh_c |
|  |  |  | ENO | 2pg_c --> h2o_c + pep_c |
|  |  |  | TPI | dhap_c --> g3p_c |
| 16 | 3.27 | 4.77 | PGK_r | 13dpg_c + adp_c --> 3pg_c + atp_c |
|  |  |  | PGM_r | 3pg_c --> 2pg_c |
|  |  |  | GAPD | g3p_c + nad_c + pi_c --> 13dpg_c + h_c + nadh_c |
|  |  |  | ENO | 2pg_c --> h2o_c + pep_c |
|  |  |  | TPI | dhap_c --> g3p_c |
|  |  |  | FBA | fdp_c --> dhap_c + g3p_c |

**
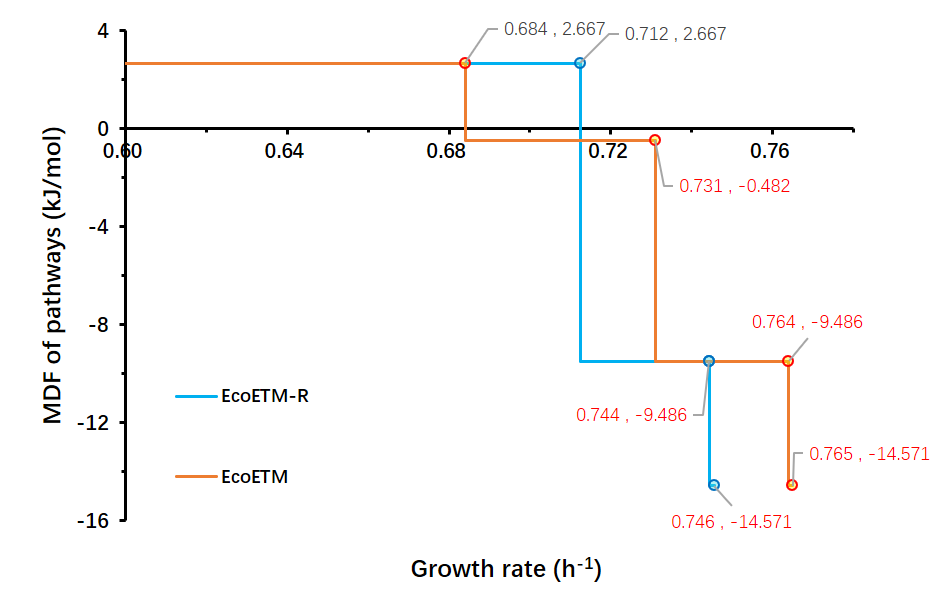
**

**Figure S6** The optimal thermodynamic driving force (MDF) of biomass synthesis pathways. The maximum yields predicted by the original EcoETM (orange line) and EcoETM-R (The revised version of EcoETM in this work, blue line) models are shown. The points where the MDF suddenly changes are circled and the coordinate values are marked. The upper bound of glucose uptake rate is set at 10 mmol/gDW/h. It can be seen that after the combination of reactions, the thermodynamically feasible yield solution space expands (from 0.684/h to 0.712/h), while the overall yield solution space shrinks (from 0.765/h to 0.746/h). The turning point where the MDF is equal to -0.482 kJ/mol disappears due to the changing of the thermodynamic feasibility of SerA (or PGCD) reaction.

**Reference**

1. Ho CL, Saito K: Molecular biology of the plastidic phosphorylated serine biosynthetic pathway in *Arabidopsis thaliana*. *Amino Acids* 2001, 20(3):243-259.

2. Mundhada H, Seoane JM, Schneider K, Koza A, Christensen HB, Klein T, Phaneuf PV, Herrgard M, Feist AM, Nielsen AT: Increased production of L-serine in *Escherichia coli* through Adaptive Laboratory Evolution. *Metab Eng* 2017, 39:141-150.
